# Enviromics and Abiotic Enviromics: Enhancing Stress Biology and Plant Resilience Breeding

**DOI:** 10.1002/advs.77304

**Published:** 2026-08-27

**Authors:** Guangchao Sun, Jose Luis Araus, James C. Schnable, Jie Xu, Henry T. Nguyen, Yunbi Xu, Yanli Lu

**Affiliations:** ^1^ Maize Research Institute Sichuan Agricultural University Chengdu China; ^2^ Yazhouwan National Laboratory Chengdu Grain and Oil Crops Innovation Platform Yazhouwan National Laboratory Chengdu China; ^3^ Plant Physiology Section Faculty of Biology University of Barcelona Barcelona Spain; ^4^ Agrotecnio CERCA Center Lleida Spain; ^5^ Department of Agronomy and Horticulture University of Nebraska–Lincoln Lincoln NE USA; ^6^ College of Agriculture Food and Natural Resources (CAFNR) University of Missouri Columbia MO USA; ^7^ National Key Laboratory of Wheat Improvement Peking University Institute of Advanced Agricultural Sciences Shandong Laboratory of Advanced Agricultural Sciences in Weifang Weifang Shandong China; ^8^ State Key Laboratory of Crop Gene Resources and Breeding Institute of Crop Sciences Chinese Academy of Agricultural Sciences Beijing China; ^9^ BGI Bioverse BGI Shenzhen China

**Keywords:** abiotic enviromics, abiotic stress, climate change, crop resilience, enviro‐epigenomics, genetic improvement, plant–envirotype interaction

## Abstract

Crop breeding faces mounting challenges from climate change, population growth, and shrinking farmland. While management and genetic improvement have contributed to yield gains, sustainable agriculture demands continuous progress to address local and future environmental stresses. Approximately 60% of past yield increases are attributed to improved abiotic stress resilience, yet further breeding advances are constrained by the complexity of environmental factors, which often co‐occur dynamically within farming systems. Enviromics—an emerging field dedicated to high‐throughput environmental characterization—enables comprehensive dissection of the “E” term in genotype × environment (*G* × *E*) interactions, improving predictive models for complex traits. Understanding how enviromics elucidates environmental effects and *G* × *E* will uncover novel mechanisms underlying crop resilience. In this perspective, we critically review the concepts of enviromics and abiotic enviromics and their impacts on plant stress responses. We further present a forward‐looking view on enviromics‐driven genetic improvement for abiotic stress adaptation, emphasizing that integrating enviromic‐assembly with multi‐omics and predictive modeling holds transformative potential for decoding molecular mechanisms and accelerating climate‐smart variety development. This framework offers a strategic pathway toward sustainable agriculture and global food security.

## Graphical abstract

Environmental stressors are captured through high‐throughput envirotyping and integrated into an enviromic framework with genomic, transcriptomic, phenomic, and environmental data. AI/ML models enable prediction and guide marker‐assisted selection, genomic selection, genome editing, and gene pyramiding toward improved abiotic stress tolerance, yield stability, and resource‐use efficiency.

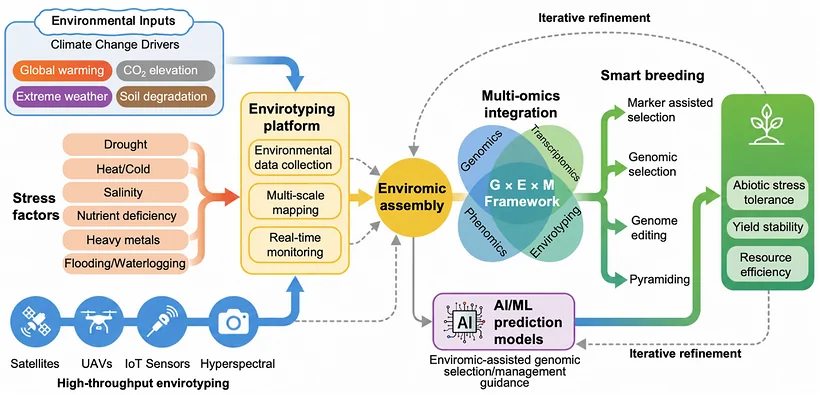

## 1 Introduction

Global agriculture faces unprecedented challenges from rapid climate change, continued population growth, and shrinking arable land. Sustaining global food security demands persistent innovation in crop breeding and field management, particularly for enhancing resilience to environmental stresses. A substantial portion of yield gains over the past century has been attributed to selection for stress tolerance and yield stability, underscoring the importance of this focus [1, 2, 3]. However, the complex and dynamic nature of environmental factors poses tremendous challenges for further breeding efforts.

**FIGURE 1:**
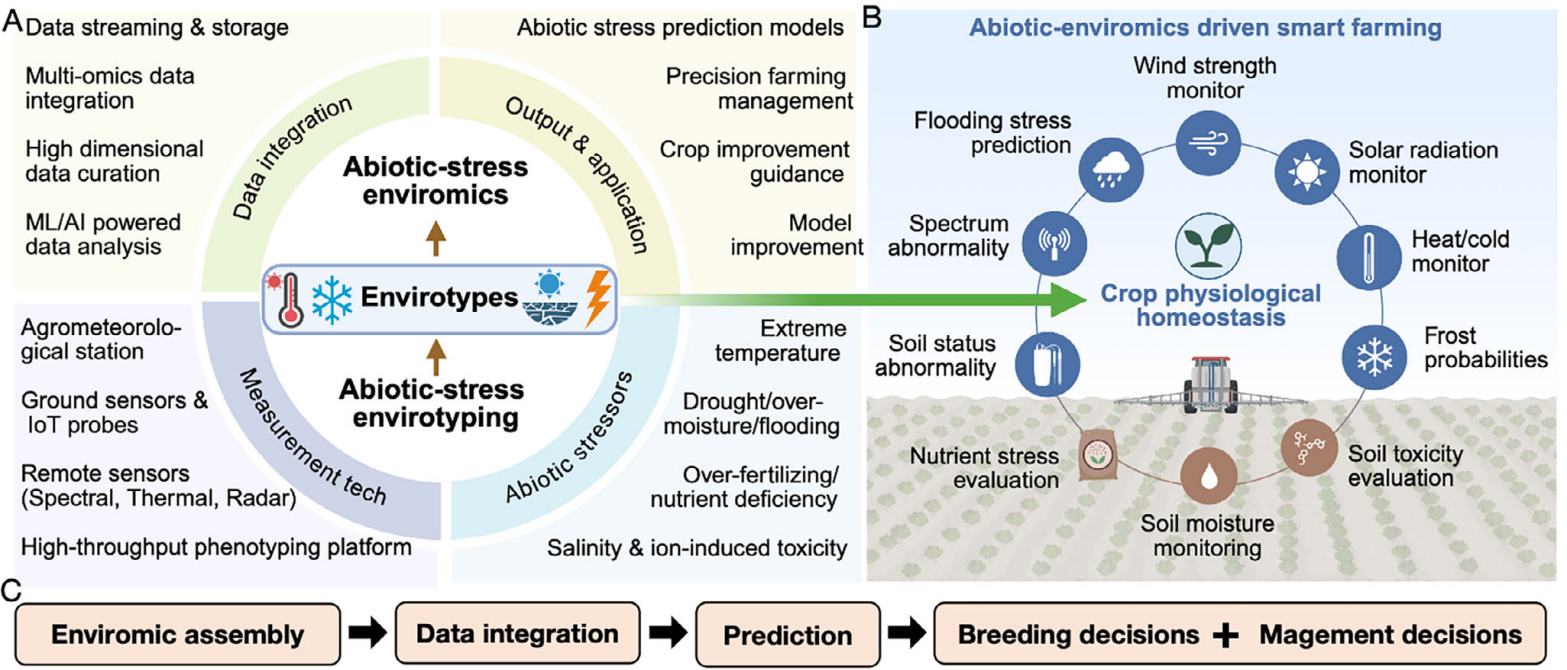
Framework for abiotic‐stress enviromics‐driven smart farming. (A) A suite of advanced sensing and imaging technologies, including satellites, drones (UAVs) equipped with hyperspectral/spectral cameras, high‐throughput phenotyping platforms, and ground‐based IoT probes and sensors, is used to quantify the abiotic stressors to which crops are potentially exposed. The high‐dimensional data generated by these sensing and measuring technologies will be streamed and stored in a high‐performance computer cluster or cloud computing platform, which runs a stable workflow or an AI‐powered data processing pipeline to integrate the multi‐omics data and perform dimension reduction. The processed data, represented as representative features, will be applied to build predictive models for a wide range of abiotic stresses, including extreme temperatures (heat/cold), drought, over‐moisture/flooding, nutrient deficiency/over‐fertilizing, and heavy metal toxicity. Such measurements, data processing, and model refining for abiotic stress prediction run in constant circles for self‐reinforcement. (B) This mode of action of abiotic‐stress enviromics forms a decision‐assisted system that powers precision farming management in which a whole range of environmental factors affecting crop physiological homeostasis can be precisely monitored, and the risk of these environmental factors becoming stressors can be assessed to provide crop improvement guidance. (C) Schematic illustration of enviromics‐assisted breeding and management pipeline: local enviromes assembled using the techniques outlined in (A), followed by iterative model refinement to inform breeding and field management decision making.

Rapid advances in both remote and proximal sensing technologies are enabling more environmental factors within a farming system to be accurately profiled, allowing a comprehensive dissection of the “E” term within the genotype × environment interaction (*G* × *E*) framework (Figure 1A). Envirotype represents a specific set of environmental variables relevant to plant growth, including soil composition, local climate features, and biotic interactions, and collectively, is defined as envirome [4].

The envirome integrates the total abiotic and biotic envirotypes driving plant adaptation, providing a framework for understanding plant physiological dynamics and developing predictive models for crop performance [5]. When one or several enviromic variables deviated from the range that crops maintain physiological homeostasis, stress responses are triggered and therefore, establishes an abiotic enviromic‐associated stress response (Figure 1B, Table 1). By applying enviromics principles and technologies to systematically characterize and quantify environmental variables associated with plants, an envirome can be assembled. Through subsequent data integration and modeling, enviromic markers can be identified to guide breeding and management decisions for crop varieties with enhanced adaptability (Figure 1C). The significance of enviromics has been acknowledged since the application of quantitative genetics to crop breeding as the “E” term in linear models. Historically, however, the “E” was often represented by a simple location or season descriptor, largely neglecting environmental factors that significantly impact phenotypic variations through interactions with genotype. By contrast, enviromics‐based yield prediction, which incorporates quantitative measurements of environmental variables, non‐additive genetic effects and *G* × *E* from multi‐environment trial, exhibited significantly improved accuracy [6]. This systematic and multi‐omics‐level approach, which integrates quantified environmental measurements in the field and molecular responses in crops, holds promise for advancing innovative and climate‐resilient breeding (Figure 1C).

**TABLE 1:** Representative envirotypes, typical thresholds, corresponding enviromics markers, and references.

Envirotype	Typical threshold	Enviromics marker	Refs.
Shading/Low light	R:FR ratio < 1.0; reduced PAR (e.g., <50–150 µmol m^−2^ s^−1^ depending on crop stage)	PHYB/PIF and CRY signaling; shade avoidance; reduced chlorophyll, photosynthesis, stomatal conductance (𝑔_𝑠_), LAI, NDVI/PRI, and SIF.	[13, 14, 15, 16]
Heat/Heatwaves	Transient temperature spikes >35 °C to 40 °C (crop‐ and developmental stage‐dependent); heat during anthesis is particularly damaging.	ROS, HSF/sHSP activation, reduced photosynthesis, increased photorespiration, accelerated senescence, membrane damage, elevated canopy temperature, reduced SIF/PRI.	[17, 18, 19, 20, 21]
Cold	Chilling: 0 °C to 15 °C (species dependent); freezing: <0 °C.	ICE1–CBF–COR activation, ROS accumulation, membrane damage, stomatal closure, reduced photosynthesis, decreased NDVI, delayed phenology.	[22, 23]
Excessive precipitation humidity drought	>800 mm (maize); >1621 ± 34 mm (rice); >450 mm (wheat) RH >60–70% >5 months (wheat/soybean); >1 month (rice/maize)	Osmotic adjustment, ROS accumulation, oxidative damage, stomatal closure, wilting, adaptive root formation, oxygen retention mechanisms, survival strategies, and reduced photosynthetic performance.	[24, 25, 26, 27, 28]
Salinity	Soil electrical conductivity (ECe) >4 dS m^−1^ (moderately saline soil).	Na^+^ and Cl^−^ toxicity, osmotic stress, ABA and ROS responses, Na^+^/K^+^ imbalance, SOS pathway activation, chlorosis, premature senescence, reduced photosynthesis, altered spectral reflectance.	[29, 30, 31]
Flooding/Waterlogging	Soil saturation for >24–48 h (species and growth stage dependent).	Hypoxia/anoxia, ethylene accumulation, aerenchyma formation, adventitious root development, reduced aerobic respiration, root decay, chlorosis, reduced photosynthetic efficiency, increased canopy temperature, altered spectral indices.	[25, 32, 33]
Nutrients	Optimum N∶ 160–332 kg ha^−1^; Optimum P ∶ 200–332 kg ha^−1^ (crop dependent) Optimum K ∶ 90–225 kg ha^−1^	Membrane and organelle damage, chlorosis and senescence, altered growth patterns, oxidative stress, and impaired stress adaptation.	[34, 35, 36]
Ozone pollution	Local excessive O_3_ loads; PM2.5 and PM10 vary by location.	Oxidative damage; foliar anatomic abnormalities: assimilatory tissues contain elevated tannin or polyphenolic compounds; increased calcium oxalate; alternated transfusion parenchyma.	[37, 38, 39]
Soil	Acidity; alkaline; heavy metals; compaction.	Salt Overly Sensitive pathway, the abscisic acid pathway, reactive oxygen species, and osmotic stress signaling; misfolded protein	[40, 41, 42, 43, 44]
Radiation stress	Ionizing radiation; anthropogenical contamination.	Chronic stress for plant and associated microbiota.	[45, 46]

**FIGURE 2:**
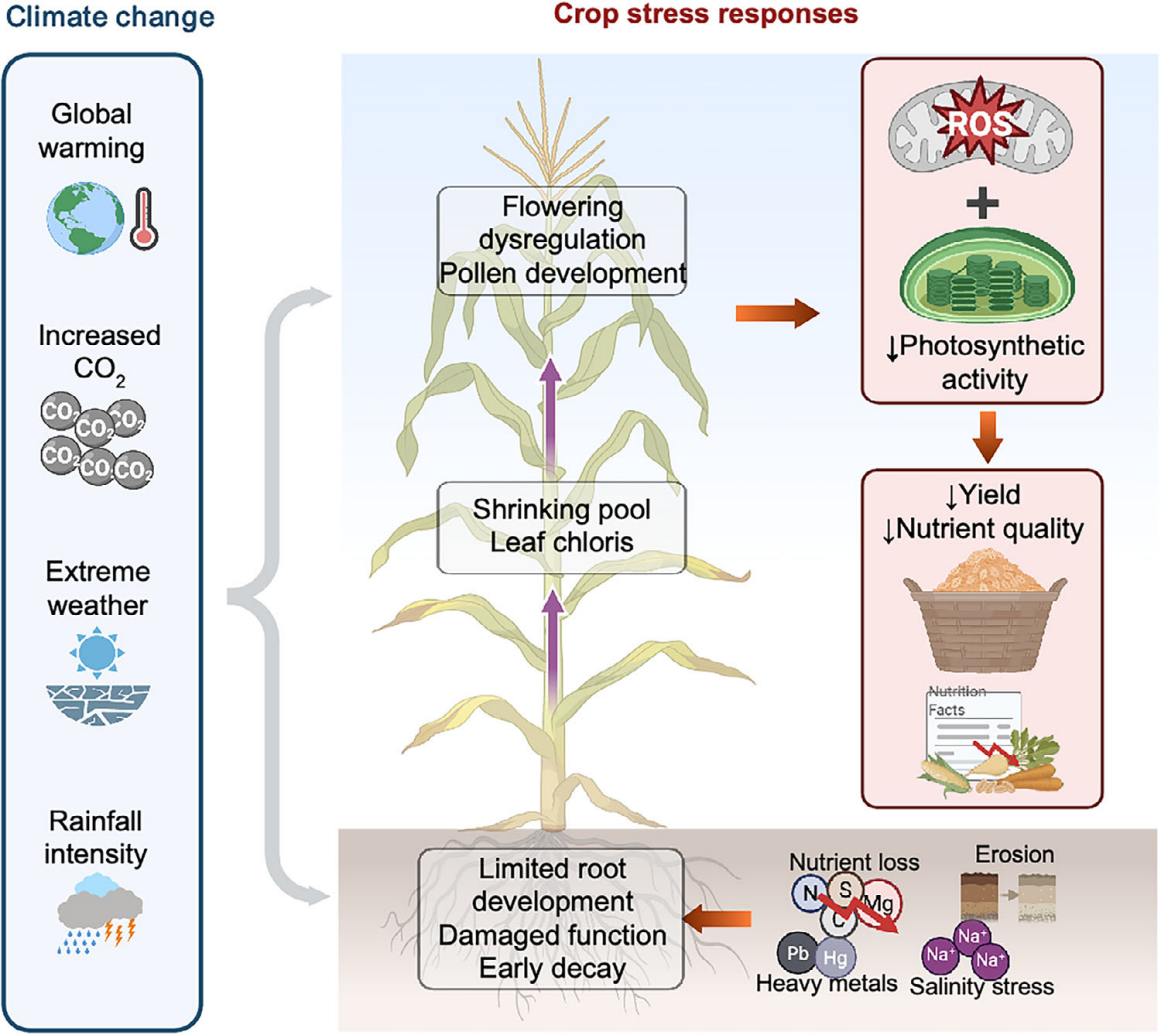
The impact of climate change on crops: Drivers, abiotic stresses, and physiological responses. Typical characterizations of climate change that significantly affect agriculture include global warming, increased atmospheric CO_2_, more frequent extreme weather events, and more unpredictable rainfall intensity, which play as key adversaries that disrupt the critical physiological homeostasis of crops. Soil deterioration due to heavy metal contamination, erosion, high salinity, and nutrient loss greatly limited root development, which directly shrinks the nutrient pool to support above‐ground vegetative development; leaf chlorosis caused by chloroplast reduction would reduce photosynthetic activity and eventually cause reproductive defects such as unsynchronized flowering. Along with yield loss, both nutrient content and nutrient composition will be significantly impaired.

The urgency of developing abiotic enviromics‐assisted breeding is underscored by the impacts of global climate change. Between 1991 and 2021, production losses in crops and livestock from all disaster events were valued at $3.8 trillion, averaging at $123 billion per year [7, 8]. Global surface temperatures during 2011–2020 were 1.1 °C higher than the 1850–1900 baseline, and this increase is projected to reach 1.5 °C in the near future driven by sustained carbon dioxide emissions from industrial and agricultural productions [9] (Figure 2). Although CO_2_ is essential for photosynthesis, excessive concentrations can induce ROS burst and reduce photosynthetic activity, ultimately diminishing productivity and quality of major crops (Figure 2). C_4_ crops such as maize exhibit remarkably reduced pollination efficiency and kernel numbers per ear leading to 84–100% yield loss when nighttime temperatures exceed 25 °C [10, 11]. Crop quality is also negatively affected when grown in environments with elevated CO_2_ concentrations (546–586 ppm), for example, it leads to 5–10% loss in protein, zinc and iron content in wheat and rice [12] (Figure 2, Table 1).

Rising global temperatures further disrupt atmospheric circulation, leading to more frequent abnormal precipitation and flooding that threaten 12–14 million hectares of farmland, accounting for approximately 30% of India rice‐growing area [47]. In addition, sea level has been projected to increase by 0.19 to 0.23 meters relative to the 1995–2015 baseline, accelerating sea water intrusion and intensifying soil salinization, which deteriorates approximately 2 million hectares of croplands annually [48, 49]. Ion toxicity and nutrient imbalance often accompany soil salinity, leading to 10–50% yield reduction in rice, wheat and other crops [50, 51]. Currently, soils containing excessive salt accounts for approximate 20% of irrigated farmlands, and this proportion is expected to increase to over 50% by the year of 2050 [52]. Furthermore, heavy metal contamination—cadmium, for example, often found in heavily industrialized regions disrupts nitrogen fixation by soybean, leading to losses of nutrient uptake by up to 50% [53] (Figure 2). Collectively, these soil‐associated stressors severely impair root system development, nutrient uptake efficiency, and induce early decay (Figure 2). A dysfunctional root system reduces nutrient pool for shoot growth and induce leaf chlorosis, thereby resulting in reproductive dysregulation such as unsynchronized flowering and non‐viable pollens (Figure 2). These extreme climate events are progressively reshaping environmental conditions and associated envirotypes in many well‐established ecosystems and agricultural systems.

Most studies on plant abiotic stresses have been conducted in controlled greenhouses or growth chambers that rarely translate to field conditions, the effects of multiple stresses co‐occurring stresses can interact in unpredictable ways, making it inaccurate to model individual stresses independently and assume additive effects in the field [54, 55, 56]. There is thus an urgent need for comprehensive models that evaluate multi‐layer, multidimensional envirotypes to develop effective strategies for mitigating the negative impacts of abiotic stresses. Gaining deep insights into the biological mechanisms and enviromic drivers underlying plant stress response is essential for both researchers and breeders.

This perspective focuses on abiotic enviromics and its applications in enhancing stress biology and resilience breeding through the development of enviromics‐based strategies including enviromics–epigenomics interplay, environmental stress management, and integrated enviromic breeding approaches. Environmental stresses evolve dynamically, the once‐benign stressors may become significantly harmful as a result of climate change and human activities (e.g., global communications). Continuous environmental sensing, real‐time stress evaluation, and plant response monitoring can provide flexible breeding and management strategies to prepare for such transformations [6]. As climate change increasingly threatens global agriculture, the importance of enviromics‐assisted breeding and farming is gaining recognition worldwide. To this end, an international consortium should be established to launch a proactive, long‐term enviromics initiative aimed at predicting dynamic abiotic stresses and delivering intelligent management strategies tailored to diverse local cropping systems.

## 2 Abiotic Enviromics: A New Enviromics Field

Abiotic enviromics is founded on the premise that plant growth and development are governed by a complex interplay of environmental factors that collectively constitute an “abiotic envirome.” These factors—including soil moisture, soil salinity, temperature regimes, nutrient availability, and atmospheric composition—dynamically shape the environmental profile a plant is associated with throughout its life cycle (Figure 1A). Like omics profiles, evirome assemblies vary in their elemental compositions. When one or more elements within a local envirome assembly amplify to deleterious levels, plant physiological homeostasis is disrupted, which hallmarks the transition from a supportive abiotic envirome to a stress‐inducing one.

### 2.1 Abiotic Enviromics‐Driven Agriculture vs. Traditional Precision Agriculture

Although abiotic enviromics shares certain methodological features with the environmental sensing frameworks in precision agriculture, it represents a distinct paradigm. Environmental sensing in precision agriculture typically focuses on optimizing inputs (e.g., water and fertilizer) for current field conditions. In contrast, abiotic enviromics aims to take environmental factors as an omics profile, in which the expression level of each elements can be modeled alongside other crop multi‐omics data. This approach seeks to uncover specific associations between environmental variables and phenotypic changes, thereby moving beyond descriptive environmental monitoring toward a predictive and integrative framework that explicitly dissects the “E” term in *G* × *E* and *G* × *E *× *M* (Figure 1B). The goal is not merely to characterize the environment, but to understand how specific envirotypes elicit particular plant responses and why different genotypes respond differently.

Furthermore, abiotic enviromics leverages multi‐platform, high throughput sensing technologies (satellites, unmanned aerial vehicles‐UAVs, unmanned ground vehicles‐UGVs, and in‐field IoT sensors) to capture environmental data at an unprecedented spatiotemporal resolution (Figure 1A). This enables the characterization of microclimates and soil conditions relevant to individual plant performance within breeding plots, rather than relying solely on regional averages (Figure 1B). Hyperspectral imaging, for instance, can detect nuanced spectral signatures indicative of subtle physiological stress that coarser‐resolution methods might overlook [57]. Consequently, the primary output of abiotic enviromics is not merely environmental maps, but quantified, multidimensional envirotypes that are directly integrated with genomic and phenotypic data. This integration facilitates the development of sophisticated genomic–enviromic prediction models that forecast genotype performance under specific enviromes, enabling targeted selection of enviromic markers in resilience breeding (Figure 1C). This forward‐looking orientation further distinguishes abiotic enviromics from precision agriculture, which emphasizes optimization of current conditions; enviromics, by contrast, informs future breeding decisions and the design of crops better adapted to dynamic environments [5, 6, 58].

### 2.2 Integrating Management (*M*) Into the *G* × *E* × *M* Abiotic Enviromics Framework

*G* × *E* × *M* framework offers a holistic perspective on crop performance. While *G* × *E* interactions have long been recognized and incorporated into association studies, enviromics explicitly acknowledges the critical role of the management (“*M*”) dimension. In abiotic enviromics, “*M*” represents the diverse agronomic practices that can either mitigate or exacerbate abiotic stresses and interact with both genotype and environment. Envirotyping cropping systems under different management regimes, for instance through IoT‐enabled soil probes like *NutriNet* or water availability sensors, enables the identification of management‐associated enviromic signatures (Figure 1C). This approach helps disentangle stress signals that originate from management practices from those arising solely from environmental factors. Moreover, by predicting the frequency and intensity of abiotic stresses, enviromics accelerates the development of adaptive management strategies (Figure 1C). This may involve selecting stress‐tolerant cultivars for forecasted harsh conditions or adjusting planting dates to avoid peak heat waves. The integration of enviromics therefore enhances not only genetic improvement but also informs dynamic, responsive management practices that optimize crop resilience. Future enviromics models will increasingly integrate management options as variables to predict optimal *G* × *E* × *M* combinations for specific agro‐ecosystems (Figure 1C).

## 3 Abiotic Stress Enviromics and Its Role in Resilience Breeding

Abiotic stresses arise from dynamic interactions between plants and their surrounding environments. Key abiotic stressors include drought, extreme temperatures, salinity, heavy metals, nutrient deficiencies, and nutrient imbalances, each of which is modulated by other environmental factors/enviromic‐variables such as soil composition, microclimate, and atmospheric CO_2_ levels, atmospheric temperature—that collectively shape the stress landscape a plant encounters [59] (Figure 2). Among the most critical factors limiting crop fitness are nutrient deficiencies, particularly nitrogen (N), phosphorus (P), and potassium (K). They can escalates into major abiotic stressors as a result of soil degradation and unsustainable farming practices (Figure 2). Nitrogen deficiency restricts leaf expansion and intrinsic photosynthetic capacity, thereby reducing canopy‐level photosynthetic efficiency. Phosphorus deficiency, in turn, impairs nutrient foraging and fixation by disrupting arbuscular mycorrhizal symbiosis and root elongation [60]. In wheat, potassium deficiency compromises stomatal regulation and osmotic adjustment, synergistically weakening drought resistance [61]. It is also important to recognize that abiotic stress can impact plants directly through composition and interactions with their associated microbial communities [62, 63].

### 3.1 Unraveling Abiotic Stress Factors Through Enviromics Approaches

In the context of enviromics, abiotic stressors can be measured by envirotyping (Table 1) and treated as an integrated component shaping *G* × *E* × *M* landscape. Profiling potential stress sources is achievable using a suite of envirotyping tools including sensors and satellites equipped with cameras, UAVs, UGVs, and other platforms widely applied for phenotyping. For instance, nitrogen deficiency in maize can be diagnosed using hyperspectral imaging data processed to calculate the Normalized Difference Red Edge (NDRE), an index that correlates strongly with chlorophyll content [64]. Numerous other vegetation indices can be derived from hyperspectral, multispectral and RGB imagery. In horticultural systems, Raman spectroscopy enables early detection of nitrogen‐deficiency phenotypes in Pak Choi and Choy Sum, facilitating non‐invasive nutrient monitoring [65]. Complementing these sensing approaches, multi‐omics integration—encompassing transcriptomics, proteomics, metabolomics, and ionomics—provides system‐level insights into the physiological consequences of abiotic stress. For example, ionomic analysis in soybean revealed that iron deficiency systematically elevates *GmFRO2* and *GmIRT1* expression, both of which are critical for rhizosphere acidification and Fe^3+^ reduction [66]. Furthermore, primary metabolomics profiling in maize, sorghum and their wild relative seashore paspalum (*Paspalum vaginatum*) identified trehalose as a potential autophagy activator that mitigates phosphorus and nitrogen deficiency stress [3]. Abiotic stress factors are crop‐specific and developmentally stage‐dependent, underscoring the need for diverse enviromic approaches to accurately identify and characterize their stress profiles.

### 3.2 Enviromic Markers as Indicators of Abiotic Stresses

Certain environment‐sensitive biochemical or physiological traits in plants can serve as enviromic markers, bridging environmental data with biological responses and serving as proxies for stress severity and plant resilience. These markers can be leveraged for major abiotic stresses and measured accurately using advanced envirotyping technologies (Table 1). For example, organic acids such as citrate and malate in root exudates can be used to indicate early phosphorus deficiency while root allantoin content has been proven to be a sensitive indicator for early nitrogen deficiency. Notably, the expression levels of genes involved in the metabolic pathways of these compounds correlate closely with quantitative measurements obtained via mass spectrometry (LC‐MS). Therefore, quick and cost‐effective transcriptomics strategies can be used to profile these abiotic molecular markers [3, 67].

Although nutrient deficiency symptoms are often straightforward to score visually in the field, precision agriculture and breeding programs increasingly rely on more accurate remote sensing approaches. For instance, near‐infrared reflectance (NIR) at 1450 nm has been shown to indicate leaf water and nitrogen content in wheat [68, 69]. Similarly, ionomic profiles, such as shoot magnesium‐to‐calcium (Mg/Ca) ratios can be used to predict magnesium deficiency in acidic soils [70]. As envirotying technologies continue to advance, enviromic profiling can be achieved at finer granularity, and the repertoire of enviromic markers will expand substantially. These markers can then be incorporated into the model *P* = *G* + *E* + *M* + *G* × *E* +* G* × *E* × *M* [4].

Machine learning approaches are particularly well‐suited for this context, as they can train phenotype prediction models on multiomics datasets to prioritize markers with strong predictive power. For example, random forest algorithms identified root cortical aerenchyma as an enviromic marker for nitrogen‐use efficiency in sorghum, linking anatomical traits to soil nitrate availability [60]. Ultimately, enviromic markers must be developed for specific crops, growth stages, and stressors, with careful consideration of throughput, affordability and end‐user acceptance.

### 3.3 Enviromics‐Driven Experimental Design in Abiotic Stress Envirotyping

Effective enviromics data collection for crop abiotic stress studies requires well‐planned, long‐term experimental layout and infrastructure capable of supporting multi‐location and multi‐trial research projects. *NASA POWER* (Prediction of Worldwide Energy Resources) offers agroclimatological and geographical data collected from 380 satellites at hourly intervals [71]. Temperature, precipitation, solar radiation and moisture measurements can be retrieved via application programming interfaces (APIs) such as those implemented in the *EnvRtype* R package, which also provides a systematic workflow to stratify enviromics data and integrate them with phenotypic and genomic data to train genomic selection (GS) models using various kernel approaches (Gaussian Kernel, Deep Kernel, etc.) and genetic architectures (additive, dominance, and *G* × *E*) [58]. Similarly, the African Soil Information Service (AfSIS) maps soil nutrient gradients across sub‐Saharan Africa regions, enabling targeted evaluation of nutrient‐deficiency tolerance [72]. At finer scales, hyperspectral imaging has demonstrated strong predictive power for foliar N, P, K, and S content of lettuce plants grown in hydroponic systems [73]. Once the target variables are defined, more cost effective multi‐spectral system can then be deployed for routine nutrient deficiency diagnosis across various crops. In addition, IoT‐enabled soil probes, such as *NutriNet* provide real‐time soil NPK measurements to guide fertilizer interventions in precision agriculture [74, 75] (Figure 1A,B). It is also critical to consider crosstalk among abiotic stresses, as disruption of metabolic or oxidative homeostasis by one stressor can lift sensitivity to other abiotic stresses in crops. For instance, zinc deficiency exacerbates oxidative damage under drought in rice by impairing superoxide dismutase (SOD) activity [76].

When designing an abiotic stress envirotyping strategy incorporating a designated set of envirotyping probes for field applications, three principles must be considered to guide the experimental setup. First, experimental conditions must be consistent and well‐controlled so that observed variations can be confidently attributed to the target abiotic stress. Second, both sensitive and tolerant genotypes should be included as reference controls. Third, two near‐iso environmental conditions only differ in the presence and absence of the given abiotic stress should be established [4]. These prerequisites enable the collection of paired envirotyping data under normal and stressed conditions, from which a relative stress tolerance index can be derived:

(1) $$Relative\;stress\;tolerance=\frac{Trait_{normal}-Trait_{stress}}{Trait_{normal}}$$

Most existing abiotic stress trials have been conducted exclusively under stress conditions with the assumption that plants grow and develop normally in the absence of stressor. In reality, however, genetic and enviromic variations already exist among test genotypes before stress is applied, moreover, envirotyping is often performed in two contrast environments (normal vs. stress conditions), yet data analysis frequently treats two conditions separately, overlooking the relative phenotypic plasticity that underpins stress tolerance. Addressing this gap will be essential for fully leveraging enviromics in resilience breeding.

### 3.4 Enviromics‐Based Prediction of Abiotic Stresses

The impact of global climate change on farming systems has been intensified over recent decades. Even when local environmental factors appear to shift mildly, their cumulative and persistent changes can foster nascent abiotic stresses within a farming system [77, 78]. For example, elevated temperature accelerates crop cycle and soil moisture loss, as a result, drought stress and substantial yield losses in wheat, rice, and maize occur more frequently [17, 79]. Thus, developing long‐term enviromics profiling platforms and data processing platforms is essential for developing a comprehensive enviromics‐based prediction (EBP) model which is capable of addressing growing meteorological extremes.

A significant advance toward this goal is Enviromic‐aided Genomic Prediction (E‐GP) framework. Different from traditional genomic prediction (GP) models, E‐GP deploys an enviromic assembly approach that uses a core set of markers to quantify environmental relatedness and incorporates this information into whole‐genome predictions for yield plasticity. This approach has been shown to outperform benchmark GP models, particularly when training sets are small [58]. In a complementary approach, enviromic assembly can be achieved by engineering a large number of environmental parameters across multiple trials for GIS‐GE based modeling. When applied to maize hybrid yield prediction, this strategy outperformed all baseline and kernel models and successfully identified the best geographically adapted genotypes or parental combinations [80]. Both frameworks demonstrate that modeling crop phenotype or yield plasticity with enviromic assemblies improves accuracy more effectively than relying solely on multi‐environment phenotyping. This enhanced predictive power also underpins the prioritization of abiotic stress‐responsive genes (CSRGs) and mechanisms with greater yield significance, thereby accelerating breeding for high‐resilience, high‐yield crops (Figure 1C).

### 3.5 Environmental Pollution and Food Quality Reduction

Beyond the direct interaction between crops and environmental factors, industrial development and other human activities have caused a range of pollution issues, such as tropospheric ozone, heavy metal contamination in soils, and waterborne pollutants from over application of fungicide, pesticide, herbicide, and fertilizers (Figure 2). These forms of pollution chronically affect crops and often accumulate in harvested organs, leading to severe food safety concerns and compromising their nutritional quality (Figure 2). One of the most severe cases is the accumulation of cadmium and arsenic in rice grains, which poses significant health risks to consumers [81]. Furthermore, modern farming systems remain heavily reliant on preventive chemicals to avoid biotic damages, and over‐application becomes increasingly prevalent. Chemical residues in crops not only reduce edibility and marketability but also undermine consumer trust. In this context, sustainable crop protection which can be enhanced by enviromics tools to improve disease and pest resistance offers a promising solution. Moving forward, enviromics strategies should be established to monitor and predict various environmental pollutants that may compromise food quality, even when they do not directly affect yield (Figures 1 and 2).

## 4 Impacts of Enviromics on Abiotic Stress Biology

Enviromics dissects detailed profiles of micro‐ and macroenvironments through large‐scale envirotyping. By providing haplotype recommendations with predictive extrapolation at a site‐specific level, enviromics therefore could direct abiotic stress research or management decisions across a wide range of conditions [80]. Plants experience continuous environmental fluctuations through their life cycle and often come across abiotic stresses. Plants response to these stressors are dynamic and complex, involving both reversible and irreversible changes. Such responses have been studied at physiological, biochemical, cellular, and molecular levels, revealing intricate networks that mediate abiotic stress adaptation. Moreover, the intensity and duration of stress, whether acute or chronic, can significantly impact the complexity of these responses [82]. Enviromics‐driven precision agriculture that features data‐driven field management holds promise for mitigating productivity losses caused by these stressors. In the long term, as artificial intelligence and computational capability continue to advance, enviromics data, multiomics and genomics–enviromics interactions can be integrated into an optimized model to support resilience breeding (Figure 1).

### 4.1 Harnessing Envirotyping to Identify Key Factors Involved in Abiotic Stresses

Typically, sources of phenotypic variation in plant populations are quantified by partitioning variance into three categories: G (genotypic variance), E (envirotypic variance) and GEI (genotype by envirotype covariance). Many studies focus primarily on mapping markers associated with G, whereas identifying markers linked with GEI remains challenging due to its inherent complexity and limited statistical power of mixed linear models to estimate these effects precisely [83]. The complexity of GEI arises from the vast pool of environmental factors that independently or together change plant physiology throughout the crop cycle. Therefore, prioritizing such factors that impose the most significant effects on physiology homeostasis by incorporating multi‐omics data with envirotyping technologies can improve genotype–phenotype association models, particularly for critical abiotic stress variables (e.g., drought, salinity, and temperature extremes). For instance, high‐throughput phenotyping platform (HTP) implementing RGB imaging have been used to assess differential yield responses under water stress [84], low nitrogen [85], high temperature [86], and pathogen infections [87]. In addition, Radar and LiDAR systems extend these capabilities with expanded measurements including root characteristics, plant architecture under the canopy and soil humidity, thereby facilitating the dissection of environmental factors imposing major effects on plant growth or agronomic traits [57]. Furthermore, enviromics can be spatially and temporally indexed in parallel with GEI effect dissection. For example, integrating photothermal time with genomic data has improved the prediction of stress‐adaptive traits, such as flowering time and water‐use efficiency [6, 88]. When certain environmental factors are identified through envirotyping alone or in combination with genotyping, they can be further validated in multi‐environment trials (METs) conducted across target mega‐environments [58, 89].

### 4.2 Physiological and Hormonal Dynamics in Crop Abiotic Stress Adaptation

**FIGURE 3:**
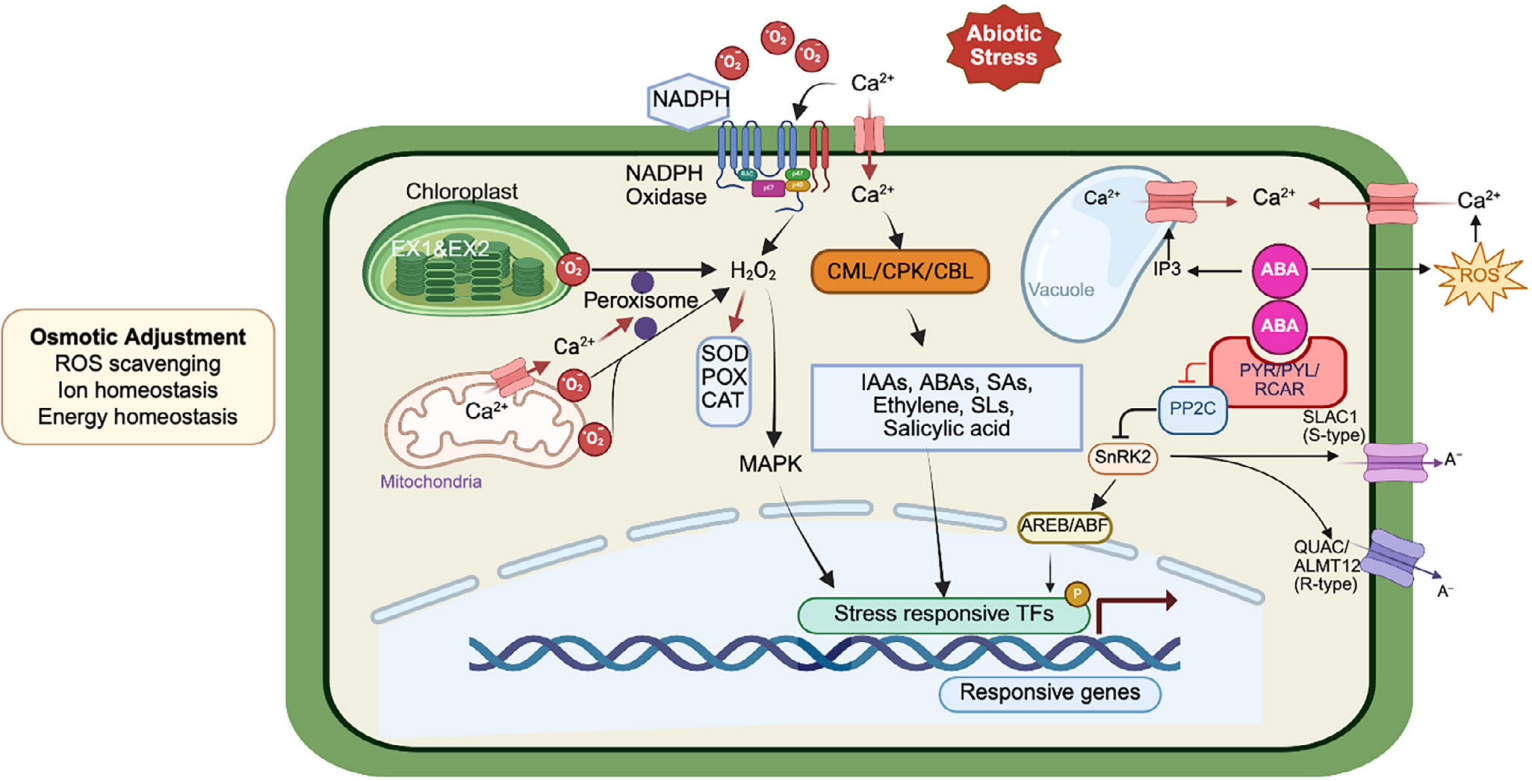
Major molecular pathways responsive to abiotic stress. This diagram illustrates the core molecular signaling pathways and metabolic adjustments triggered by abiotic stress in plant cells. Upon stress perception, and immediate influx of calcium ions (Ca^2+^) from apoplast and internal stores (vacuole, ER) activates a series of signaling pathways. The stress‐induced hormone abscisic acid (ABA) binds to its PYR/PYL/RCAR receptors, which subsequently inhibit PP2C phosphatases. This inhibition derepressed SnRK2 kinases leading to phosphorylation of AREB/ABFs to activate stress‐responsive gene expression and activation of S‐type and R‐type ion channels to promote anion efflux. Calcium signaling components, including CaMs/CMLs, CDPKs/CPKs, and CBLs/CIPKs, activate the biosynthesis of various phytohormones (e.g., ABA, IAA, ethylene, strigolactones, and salicylic acid). These hormones activate specific transcription factors to promote the expression of stress‐responsive genes. Calcium signals also activate NADPH oxidases (RBOHs) to produce hydrogen peroxide from apoplastic superoxide free radicals. It also promotes such conversion of superoxide produced from chloroplasts and mitochondria under stress. Superoxide is rapidly converted to hydrogen peroxide by superoxide dismutase (SOD) in various compartments such as peroxisome. The accumulation of hydrogen peroxide activates ROS scavengers, such as peroxidases (POX) and catalases (CAT), and can also directly activate MAPK signaling cascades. These pathways converge on stress‐responsive transcription factors. These convergences help plants to deploy three major cellular metabolic strategies including energy resource management (metabolic shifts, nutrient partitioning, and growth‐defense trade‐off); protective molecule synthesis (heat shock proteins (HSPs), late embryogenesis abundant (LEA) proteins, and detoxification enzymes) to mitigate damage from toxic compounds and osmoprotectant and antioxidant production (antioxidants (e.g., glutathione, ascorbate, and flavonoids), compatible solutes (e.g., proline, glycine betaine, and trehalose)) to maintain osmotic homeostasis.

**FIGURE 4:**
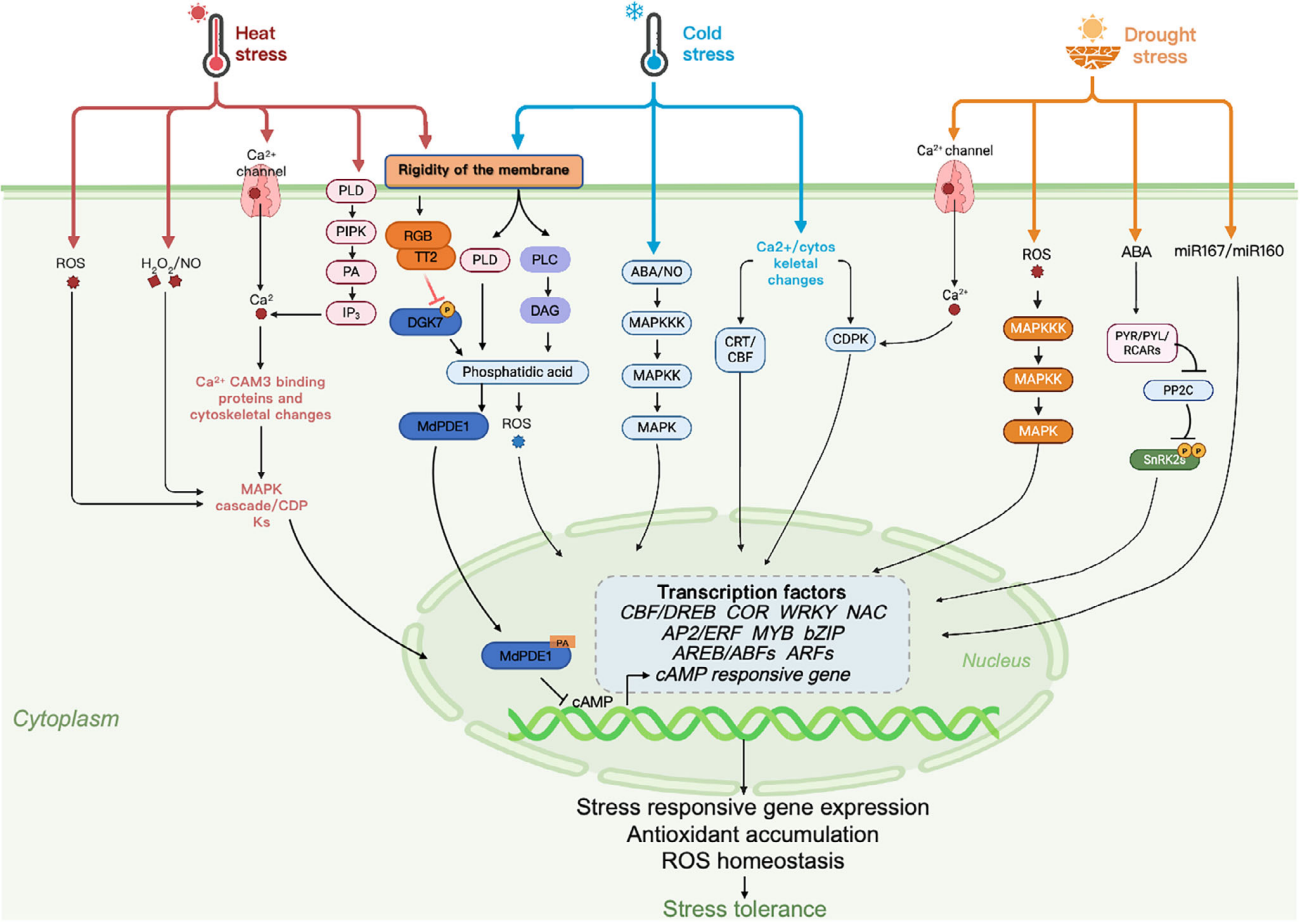
Transcription factor activation by signaling cascades in response to heat, cold, and drought stress in plants. Plants sense temperature and water availability through membrane‐based sensors that detect changes in lipid homeostasis and membrane rigidity. Perception of heat stress triggers an immediate influx of calcium ions (Ca^2+^), the production of reactive oxygen species (ROS), and the release of signaling molecules such as hydrogen peroxide (H_2_O_2_) and nitric oxide (NO). Calcium‐binding proteins (e.g., calmodulins, such as *CAM3*) are activated upon calcium influx, leading to cytoskeletal reorganization and the activation of mitogen‐activated protein kinase (MAPK) cascades and calcium‐dependent protein kinases (CDPKs). Heat stress also activates a G‐protein signaling module involving the Gγ subunit *TT2*, which negatively regulates *DGK7* activity via dephosphorylation. Upon heat perception, membrane lipid remodeling generates diacylglycerol (DAG), which is phosphorylated by *DGK7* to produce the lipid second messenger phosphatidic acid (PA). PA binds and activates *MdPDE1*, a metal‐dependent phosphodiesterase, triggering its nuclear translocation. Within the nucleus, *MdPDE1* hydrolyzes the second messenger cAMP, leading to transcriptional reprogramming and activation of heat shock factors and other thermotolerance genes. Cold stress leads to a rapid increase in cell membrane rigidity, which activates phospholipase D (PLD) and diacylglycerol (DAG) kinase, producing the lipid secondary messenger phosphatidic acid (PA). This pathway finally causes a ROS burst and activates responsive transcription factors. ABA or NO molecules accumulate upon cold stress and lead to the MAPK signaling cascade to activate cold‐responsive transcription factors. Calcium influx and cytoskeletal changes also activate the *CRT/CBF* and CDPK pathways for the activation of cold‐responsive transcription factors. Drought stress causes calcium influx that activates transcription factors via CDPKs. ROS burst activates the MAPK signaling cascade, ABA‐dependent activation of SnRK2s, and ultimately induces the expression of microRNA 167 and 160, which in turn regulate drought‐responsive transcription factors.

Abiotic stresses trigger a series of physiological and biochemical responses in crops to maintain cellular homeostasis (Figure 3). Enviromics provides a powerful lens to understand these intricate adaptation mechanisms by linking precise envirotyping with detailed molecular and physiological profiling. Upon perception of abiotic stress, plants initiate rapid and sophisticated signaling pathways to mitigate negative consequences (Figure 3). These pathways often involve an immediate influx of calcium ions (Ca^2+^) into the cytoplasm from both the apoplastic and internal stores (e.g., vacuole and endoplasmic reticulum) (Figure 3). This Ca^2+^ signal acts as a crucial secondary messenger, activating a cascade of downstream responses. Adverse environmental conditions often induce oxidative stress, featured by the overproduction of reactive oxygen species (ROS) such as superoxide (O^2+^) and hydrogen peroxide (H_2_O_2_). To counter this situation, plants accumulate osmolyte (e.g., proline and trehalose) and oxidant scavengers (e.g., glutathione and ascorbate) to protect cellular components from being damaged [5, 90] (Figure 3). Enviromic signatures correlated with oxidative stress surging can assist target breeding for improved antioxidant capacity. Beyond the above‐mentioned rapid cellular responses, plants also deploy systematic acclimation of stress through a dynamic hormone network [91]. Phytohormones such as abscisic acid (ABA), auxin, brassinosteroids, cytokinins, ethylene, and gibberellins act as key elements in abiotic stress response [92]. High salinity and drought usually elevate ABA levels to stimulate stomata closure to maintain water balance and transcription of responsive genes [93] (Figure 3). Gamma‐aminobutyric acid (GABA) acts as a signaling molecule for oxidative, salt, drought, and heavy metal stresses, it also triggers plant immune responses to pathogen infections [94]. Brassinosteroids represent a unique class of polyhydroxylated steroid hormones in plants coordinating several essential biological processes, including cell division, cell elongation, photomorphogenesis, xylem differentiation, reproduction, and stress acclimation. Strigolactones, on the other hand, are another class of plant hormones widely acknowledged for their roles in plant–microbe interaction; they are also involved in abiotic stress responses, either acting independently or through crosstalk with other phytohormone [95, 96]. Furthermore, melatonin acts as a strong antioxidant that modulates reactive oxygen species (ROS) and reactive nitrogen species (RNS) in response to abiotic stresses [97] (Figures 3 and 4). Plants perceive temperature and water availability through membrane‐based sensors that detect changes in lipid homeostasis and membrane rigidity. Upon stress perception, distinct yet interconnected signaling cascades converge on the activation of stress‐responsive transcription factors (*DREB*, *NAC* transcription factors). A recent study revealed that a G‐protein gamma subunit *TT2* functions as a brake of *DGK7* dependent PA production by suppressing *DGK7* activity via dephosphorylation. Upon heat perception, membrane lipid remodeling generates diacylglycerol (DAG), which is phosphorylated by *DGK7* to produce the lipid second messenger phosphatidic acid (PA). PA binds and activates *MdPDE1*, a metal‐dependent phosphodiesterase, triggering its nuclear translocation. *MdPDE1* hydrolyzes the second messenger cAMP in nucleus leading to transcriptional reprogramming and activation of heat shock factors and other thermo‐tolerance genes [98] (Figure 4). Cold stress also activates PLD and PLC dependent PA production, followed by ROS burst that activates cold responsive transcription factors (Figure 4).

### 4.3 Dynamic Epigenetic Responses to Environments in Plants

**FIGURE 5:**
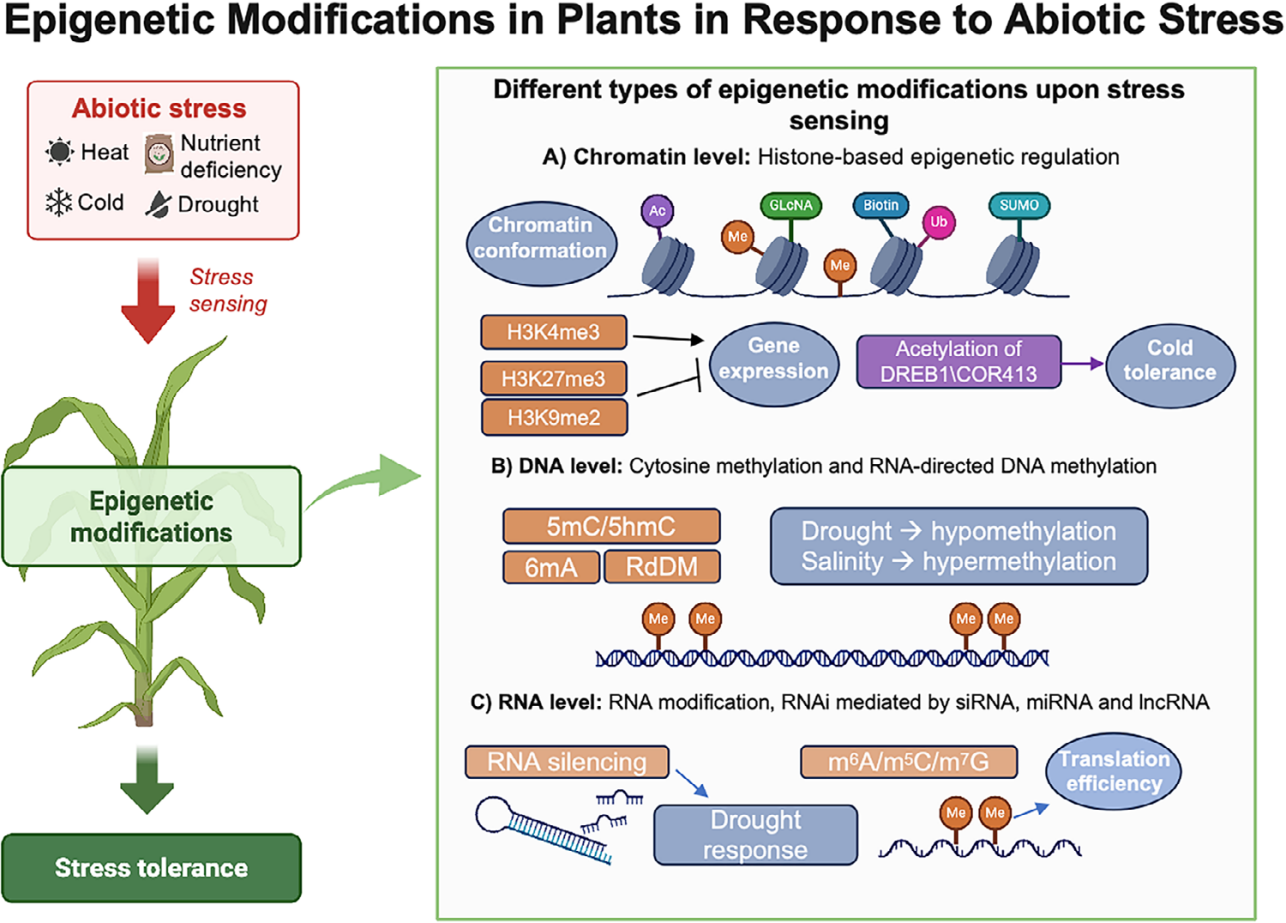
Epigenetic mechanisms regulating plant responses to abiotic stress. Abiotic stresses—including heat, cold, drought, and nutrient deficiency—trigger dynamic epigenetic modifications that alter gene expression without changing the underlying DNA sequence. These modifications occur at multiple interconnected levels: (A) Chromatin remodeling: The structure of chromatin is modified through post‐translational modifications (PTMs) on histone tails. These include acetylation, methylation, biotinylation, ubiquitination, sumoylation, and O‐GlcNAcylation. These PTMs alter chromatin architecture (e.g., promoting euchromatin [open] or heterochromatin [closed] states), thereby controlling the accessibility of DNA to the transcriptional machinery. The incorporation of specific histone variants (e.g., H2A.Z) and the recruitment of non‐histone chromatin‐associated proteins (e.g., reader proteins, chaperones) further fine‐tune the transcriptional output in response to stress signals. Collectively, these epigenetic layers form a complex regulatory network that facilitates plants to rapidly adapt to environmental challenges, maintain cellular homeostasis, and potentially transmit stress memories to offspring. (B) DNA methylation: The covalent addition of a methyl group to cytosine bases (primarily in CpG, CpNpG, and CpHpG contexts) can silence transposable elements and regulate gene expression. Stress can induce targeted changes in DNA methylation patterns (hypomethylation or hypermethylation) across the genome. (C) RNA modifications: The epitranscriptome, comprising chemical modifications to RNA molecules, plays a key role in regulating mRNA stability, splicing, and translation. Common stress‐responsive modifications include methylation of adenosine (m6A) and cytidine (m5C), as well as pseudouridination.

Epigenetic modification of nucleic acids and histones affects the expression of products encoded by genes in either a reversible or heritable way [99, 100]. Such modification is deployed in three interconnected layers, DNA, RNA, and chromatin, which have been shown to regulate gene expression through different mechanisms in response to various types of external or internal stimuli (Figure 5). The most studied DNA modifications are cytosine methylation (5mC and 5hmC) and adenine methylation (6 mA) [101, 102]. Plants also deploy a unique RNA‐directed DNA methylation (RdDM) carried out by *DRM2* to silence transposable elements, thereby maintaining genome stability [103]. At the RNA level, N6‐methyladenosine (m^6^A) is the predominant modification regulated by m^6^A writers (*MTA* and *MTB*), erasers (*ALKBH10B*), and readers (*ECT2*, *ECT3*, and *FIP37*) [104, 105, 106]. Histone methylation, varied by lysine position and degree of methylation (e.g., H3K9me2, H3K27me3, and H3K4me3), regulates the accessibility of gene transcription machinery by altering chromatin configuration [107] (Figure 5).

Epigenetic modifications are dynamic over time throughout the life cycle of organisms, and the modification landscape can change significantly in response to specific external stimuli at particular developmental stages [108, 109]. For example, temperature, light intensity, nutrient availability, and pollutant exposure provoke epigenetic changes and the variation in epigenetic response underlie phenotypic plasticity to certain environmental cues [110, 111]. RdDM mediated by small RNAs or long noncoding RNAs has been implicated to play a critical role in drought and salinity response in maize [112]. Diverged epigenetic responses to drought and salinity has been revealed in different plant species beyond the predominantly shared responsive mechanisms. For instance, drought gives rise to genome‐wide reduction of methylation level (hypomethylation) in maize [113] and rice [114] while salinity causes genome‐wide hypermethylation in barley shoots [115]. Moreover, histone acetylation of *DREB1* and *COR413* was linked to cold stress resilience in maize [116] (Figure 5).

### 4.4 Leveraging Enviro‐Epigenetic Interactions for Breeding Abiotic Stress‐Resilience

Enviro‐epigenomic interaction goes beyond correlations. By employing high‐throughput sequencing technologies, bioinformatics tools, and functional genomics approaches, researchers are now gaining deeper insights into the relationships between specific envirotypes and epigenetic modifications [117, 118, 110, 119]. Unlike genome‐wide association studies conducted between genomic variants and traits of interest, association studies deployed with genome‐wide methylation variations shed light on how genomic DNA mutations altering methylation patterns that represent epialleles of epiQTLs could affect heritable gene expression in response to environmental stimulates [120, 121]. Some epialleles, such as the peloric mutant in Linaria or the tomato ripening variant, exhibit stable meiotic inheritance over many generations, yet they remain prone to occasional reversion [122]. There has been mounting evidence showing epigenetic variations can be selected to improve agronomic traits [123]. In Arabidopsis, several flowering time and primary root length associated epiQTLs have been identified, offering potential targets for breeding stress‐resilient crops [124]. Moreover, studies in *Brassica* species showed epigenetic diversity underpins morphological variations in *Brassica oleracea* [125] and shapes key pollinator‐attraction traits such as floral scent and morphology in *Brassica rapa* [126]. Furthermore, wheat plants acquire a stress “memory” that enhances carbon assimilation and nitrogen‐use efficiency if drought stress occurred at vegetative stages, thereby mitigating yield losses when exposed to combined drought and heat stress during reproductive stages [127]. Therefore, targeting specific stress‐related genes or regulatory regions that acquire resilience conferring epigenetic modifications holds promise for stress tolerance breeding in crops [128]. These compelling examples notwithstanding, the heritability of epigenetically mediated phenotypes is subject to fundamentally distinct mechanisms—mitotically heritable memory versus meiotically transmitted epiallelic variations across generations [122]. Environment‐induced chromatin modifications such as histone acetylation and methylation at stress‐associated loci are largely reset during reproduction, thereby conferring unstable transgenerational inheritance [122]. Epigenetic integrity is particularly vulnerable in wide interspecific crosses or newly formed polyploids, where both methylation reprogramming and TE re‐activation is frequently observed [129]. These inherent limitations suggest that, while epigenetic variation offers valuable insights into stress biology and phenotypic plasticity, its direct exploitation in conventional breeding programs—which rely heavily on predictable and meiotically inheritable traits—remains a major hurdle. Future efforts to dissect the stable, regulatory epigenetic circuits that mediate GEIs will ultimately enable rational design and innovative breeding strategies to enhance crop resilience.

## 5 Enviromics‐Assisted Breeding for Abiotic Stress Resilience

**FIGURE 6:**
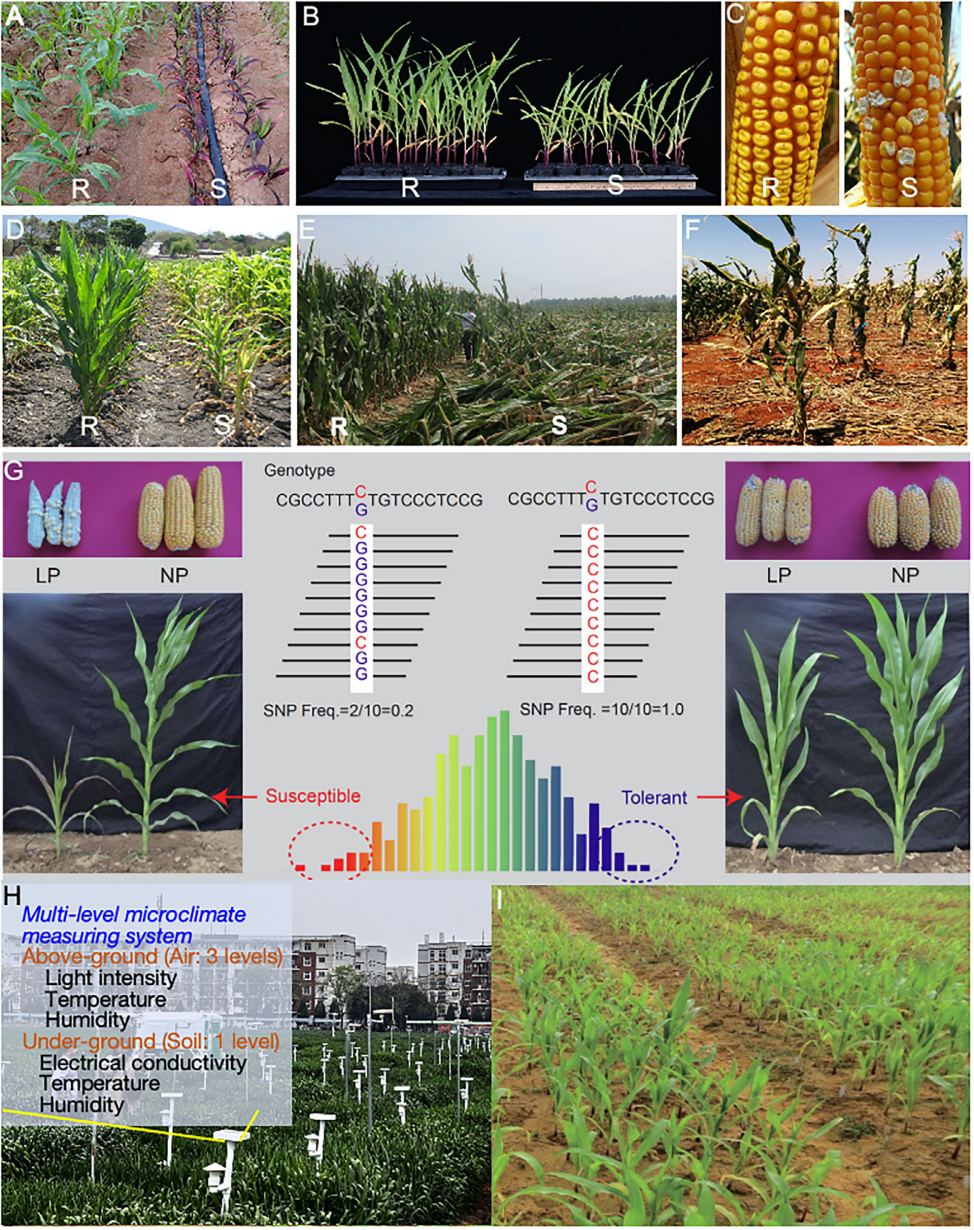
Evaluation, selection, and bulked sample analysis for abiotic stresses in maize. (A) Tolerance and sensitivity responses to low phosphorus, showing different leaf colors, with a photo taken in Sanya, Hainan, China (2018). (B) Tolerance and sensitivity response to low nitrogen, maize seedlings showing different leaf chlorosis and plant height reduction. (C) “Puffed‐kernel” observed on maize ears before harvest as triggered by “dry‐then‐wet” climate. Maize varieties may exhibit tolerance, with no puffed kernels (left), or sensitivity, with puffed kernels (right), as seen in a photo taken in Gongzhuling, Jilin, China (2015). (D) Tolerance and sensitivity responses to drought before flowering in Tlatizapan Experimental Station, CIMMYT, Mexico. (E) Plants showing resistance (standing) and sensitivity (lodging) to strong wind, with a photo taken in Taiyuan, China (2013). (F) Plants selected for drought tolerance (by water stress environment) and for other traits by marker‐assisted selection, with a photo taken in the CIMMYT Experimental Station, Kenya (2015). (G) Bulked sample analysis using low‐phosphorus response as an example. Significant phenotypic differences were observed for both plant height and seed setting. Plants with extreme phenotypes selected from the two tails were used for DNA extraction, and then the DNA bulks were sequenced to identify the markers associated with low‐phosphorus tolerance that show a significant signal difference. LP: low phosphorus; NP: normal phosphorus. (H) A multi‐level microclimate measuring system, ClimaProfile (PhenoTrait Technology Co. Ltd., Beijing, China), was set up in each plot for monitoring climate and abiotic stresses, providing all‐weather, high‐precision, three‐dimensional and time‐series environmental data by integrating hardware, sensors, communication modules and cloud platforms (the photo taken at Northwest A & F University Experimental Station, China, 2026). (I) Field screening for low phosphorus tolerance, with a photo taken in Guangzhou, China (2012).

Since the concept of envirotyping was proposed in 2016 [4], efforts have been made to advance enviromics approaches for exploring novel molecular mechanisms underlying environmental response for crop breeding [5]. However, a substantial gap remains in fully understanding the interplay between crop internal homeostasis and enviromics dynamics, particularly how crops respond to abiotic stress and how their resilience can be enhanced (Figure 6A–F). Since the 1930s, following the dramatic yield increase driven by the utilization of heterosis, maize productivity has become increasingly dependent on higher planting densities, which intensify competition for water, nutrients and radiation; as a consequence, abiotic stress tolerance is now fundamental to yield stability. With the emergence of novel envirotyping technologies, integrating enviromics with multi‐omics data into phenotype prediction model can guide rapid selection of optimal combinations and inbred parental lines, thereby enhancing the efficiency of current breeding systems (Figures 1 and 6G–I). Genomic and enviromics integration improves prediction accuracy and increases selection response in a cost‐effective manner. Breeding programs that deploy models trained with *G* × *E* interactions combined with enviromic (environmental covariate) kernels can boost prediction accuracy per dollar invested by up to 145% compared to models without enviromics data [130]. CRISPR‐Cas9 editing of stress‐related genes (e.g., OST1 kinase) further refines the precision in engineering resilience [131]. High‐throughput phenotyping platforms link these traits to enviromic variables, enabling selection for drought‐resilient root systems or heat‐tolerant photosynthesis [57]. For instance, enviromic models showcased promising prediction on maize hybrid performance under water scarcity by integrating root angle data with soil moisture profiles [132] Therefore, further research on smart breeding that leverages abiotic enviromics represents a promising strategy for developing resilient crops.

### 5.1 Envirotyping in Managed Farming Environments for Precision Crop Stress Evaluation

**FIGURE 7:**
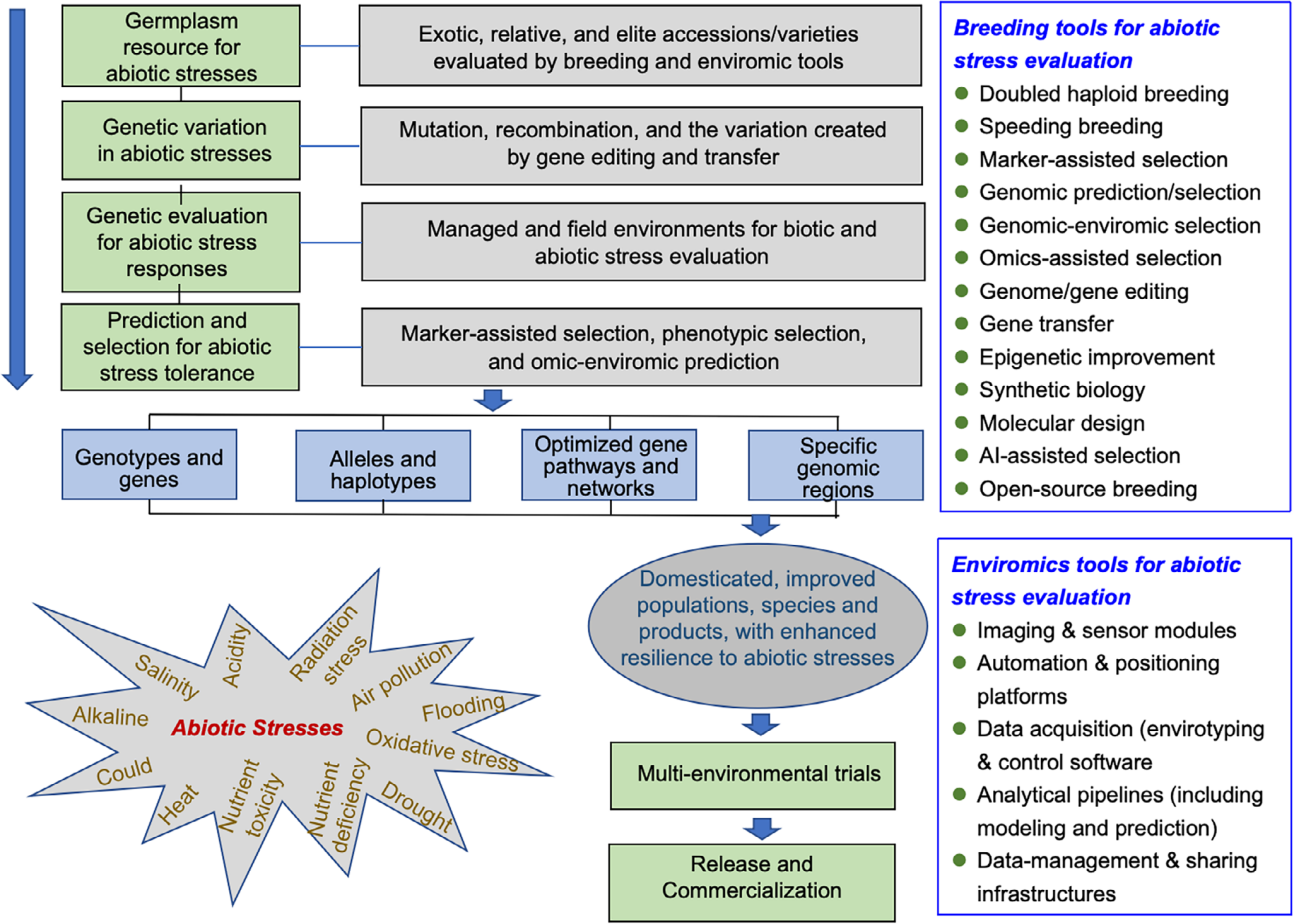
Enviromics‐assisted breeding for abiotic stress resilience. The blue arrows indicate a flowchart for the breeding procedure, from germplasm resource to variety release and commercialization for abiotic stress tolerance (light green boxes), following the associated research and experimental processes (gray boxes). Associated functional elements that are manipulated in breeding are shown in light blue boxes. Examples of abiotic stresses to be managed for resilience breeding are shown in lower left corner. Techniques and tools for breeding and enviromics involved in abiotic stresses are included in the two boxes in the right.

Envirotyping leverages a range of advanced technologies to profile environmental variables including temperature, soil moisture, salinity and meteorological measurements, thereby enabling a more nuanced dissection of environmental, management, and *G* × *E* × *M* effects (Figures 1, 6H, and 7). When these variables deviate from their optimal ranges, stress responses are elicited, diverting energy that would otherwise be allocated to biomass or yield accumulation. Accurately capturing such stress signatures, however, requires predictive or evaluative models built upon large enviromic and phenotypic datasets collected from managed farming systems [5]. High‐throughput phenotyping platforms such as PhenoLab facilitate automated imposition of controlled abiotic stress conditions and plant care regimes [133, 134]. This allows real‐time physiology monitoring across a range of stress intensities, enabling efficient identification of stress‐tolerant genoyptes [57] (Figures 1 and 7). For a given genotype, such strategies can identify enviromic signatures that give rise to abiotic stress responses across varying environments, providing actionable guidance for site‐specific management interventions to mitigate potential yield losses. For example, multi‐environment trials combined with photothermal time as an enviromic index have improved the prediction accuracy by linking genomic data with environmental covariates [6, 58]. Additionally, the Decision Support System for Agrotechnology Transfer (DSSAT) cropping simulation model has been used throughout the world in the past two decades to help field management decisions [135]. One of its modules, CRERS‐Wheat, continues to be refined and enriched as new data are incorporated, and has demonstrated reasonable accuracy for predicting yield gains under favorable conditions [136]. Models built using this framework will be continuously refined as more field data become available (Figure 1A). The inclusion of management layers further enhance their predictive capacity and practical utility for on‐farm decision‐making.

### 5.2 Integrative Breeding Strategies for Abiotic Stress Resilience

A wide variety of envirotypes can manifest as abiotic stressors that impair plant growth and productivity across diverse environments (Table 1) (Figure 6A–F). Conventional breeding typically tackles each stress individually, a process that is time consuming and labor‐intensive. To expedite breeding progress, integrative breeding strategies that combine advanced technologies are increasingly warranted (Figure 6G–I). Bulk segregant analysis (BSA) remains a widely applied approach for genomic‐resilience association identification and marker development for resilience selection (Figure 6G). Within an association panel, a small subset of population often show either highly resistant or highly susceptible to specific stresses including nutrient deficiency (Figure 6A,B), moisture fluctuation (Figure 6C), drought (Figure 6D), and lodging (Figure 6E). This extreme phenotypic divergence facilitates the inference of causal alleles segregating at extreme frequencies between the two contrasting pools (Figure 6G). Although BSA offers only coarse mapping resolution, often identifying large candidate intervals, refinement is possible through advances in experimental design and algorithm innovations [137, 138]. Once validated, markers derived from BSA can be used for fine mapping and subsequent marker‐assisted selection (MAS) of target phenotype [139, 140] (Figure 7). Unlike phenotype dependent selection, which requires exposing plants to specific stresses for a trait of interest, MAS can be conducted with DNA extracted from seeds or seedling tissues, enabling condition independent selection for tolerance to multiple stresses. Similarly, genomic selection (GS) and genomic–enviromic selection (GES) are well‐suited for complex traits governed by multiple genes or complicated metabolic pathways. A notable advancement in this context is the incorporation of environmental covariates, such as soil properties and meteorological data, into machine‐learning frameworks, which substantially enhances prediction accuracy for complex traits like drought tolerance [5, 141] (Figure 6H,I and 7). A successful case is the integration of genomic and enviromic data into kernel models for maize hybrid yield prediction, which significantly improved accuracy under water‐limited conditions [6]. Moreover, smart breeding integrates enviromic data with genomic selection and machine learning to optimize stress management and yield prediction. For instance, the CERIS‐JGRA pipeline combines environmental indices (e.g., rainfall patterns) with genomic markers to predict yield stability [65]. These approaches enable breeders to prioritize genotypes tailored to stress‐prone mega‐environments.

Gene transfer from distantly related or alien species can introduce novel abiotic stress tolerance genes or mechanisms [142], while novel genes, alleles, and their combinations could be created through genome editing [143]. At both individual and population level, novel haplotypes related to superior traits can be designed or synthesized [5, 144]. Ultimately, genes and gene combinations conferring tolerance to multiple abiotic stresses can be pyramided into a single genetic background to develop cultivars adapted to diverse or specific stress conditions. Before official release, developed cultivars should be validated under stress regimes set in controlled conditions and naturally occurring stress environments.

### 5.3 From Lab to Field: Bridging Controlled and Field Environments

Although translating genetic mechanisms of abiotic stress resilience from controlled environments to field conditions remains challenging, field validation is undoubtedly a critical step to accomplish a successful breeding cycle [145]. Considerable advances have been made in cloning genes associated with abiotic stress tolerance. For instance, *ZmRR1* [146] and *ZmCOOL1* [147] have been implicated in cold tolerance in maize while *RTN16* has been linked to maize drought tolerance [148]. Despite these promising discoveries, introgressing these beneficial alleles into elite inbred lines typically requires several generations of recombination and field selection, with the risk of reduced or loss of effectiveness across different genetic backgrounds and environments. Envirotypes, along with their interactions with genotypes, impose complex pressure that may override mechanisms deployed by genes in controlled conditions. Therefore, incorporating envirotyping with the initial experimental design for phenotyping in field environments enable more accurate and efficient assessments of plant performance under natural stress conditions. As strategies for integrating enviromics and genomics continue to improve, selection become less biased and more precise so that variations in known genes or haplotypes associated with abiotic stress tolerance can be selected and validated under natural or field conditions (Figure 7).

## 6 Challenges and Future Perspectives in Abiotic Enviromics

While enviromics‐assisted breeding framework proposed in this perspective holds immense potential to revolutionize breeding architectures, its final implementation faces substantial technical and financial bottlenecks. Addressing these challenges is essential for advancing the field toward sustainable agriculture and global food security.

### 6.1 Technical and Infrastructure Bottlenecks

Implementation and maintenance of a comprehensively integrated sensor infrastructure lies at the core of enviromics‐assisted breeding. Breeding programs rely heavily on high‐quality heterotic groups for combination selection, however, within enviromics framework, rapid phenotyping of heterotic germplasms across diverse agricultural systems must first be undertaken. Establishing the sensing network such as IoT probes, remote sensing platforms like UAVs and satellites is economically intensive. The initial investment for developing and deploying such infrastructure can be prohibitive, particularly in resource‐limited regions, where concerns about facility longevity and accessibility are often raised. Furthermore, standardizing and curating data collected from heterogeneous agricultural environments—especially when extreme conditions occur—are challenging. Robust and accurate data processing models must be developed based on stable multi‐year, multi‐location datasets, to enable precise variance partitioning to various enviromic factors and thereby inform breeding decisions [141].

### 6.2 Computational and Data Integration Challenges

Sensing network configuration in enviromics‐assisted breeding framework bring unprecedented technical challenges in data collection, integration and processing. The first hurdle lies in the sheer volume and complexity of enviromics data, which demand sophisticated computational resources and substantial storage capability. While data storage can be achieved in a static or cloud space, streaming data to an analytical space relies to a stable, fast and high‐bandwidth connections, which are often scarce resources in many under‐developed regions. Second, the high‐dimensional and multi‐variable nature of enviromics data calls for machine‐learning based processing approaches. However, data cleaning and labeling can be particularly cumbersome in the early stages, urging the need of robust analytical workflow. Furthermore, AI‐driven feature extraction typically requires electricity‐ and capital‐intensive infrastructures such as high performance computing clusters.

### 6.3 Translating Discoveries to Practical Breeding Outcomes

Translating enviromics‐driven insights to tangible crop improvements faces a series of translational hurdles. The entire pipeline starting with data collection, preprocessing, curation, labeling, modeling to subsequent genetic association studies aimed at identifying genomic loci or networks involved in phenotype establishment in specific environments is both labor‐intensive and capital‐intensive. The initial implementation of enviromics‐based crop breeding may be relatively straightforward under a controlled setup such as greenhouse or dedicated field plots. However, genetic mechanisms uncovered under these conditions often do not directly translate into field due to complex and dynamic *G* × *E* and *G* × *E* × *M* interactions. In addition, robust validation of enviromics‐derived markers and genes in diverse field conditions is paramount [145].

On the other hand, scaling enviromics for commercial breeding programs presents further challenges. The costs associated with high‐throughput phenotyping and envirotyping, multi‐omics profiling, and advanced data analytics need to must decrease substantially to become economically viable for wide spread adoption. Scalable solutions that can be applied across numerous breeding materials across diverse environments are essential [149]. While enviromic markers show great promise, enviromics assemblies must be compared across vastly different environments over multiple generations to evaluate stability of enviromic markers. Similarly, efforts in microbiome engineering—if considered in a broader context—face challenges related to the stability and predictability of microbial community manipulations under variable field conditions [150].

### 6.4 Enviromics‐Assisted Breeding

To overcome the challenges outlined above, future efforts in abiotic enviromics should prioritize several strategic directions. First, an international consortium should be established to facilitate open‐source data collecting, platform sharing, and standardized data processing, thereby reducing computational burdens and accelerating collaborative research [5, 89]. Second, affordable, robust and high‐resolution sensor technologies are needed to improve data generality, quality and spatial coverage [80]. Third, a pan‐reverse phenotyping knowledge database should be established to enable comparative enviromics to formulate a reliable stress‐phenotype association model. Finally, cultivating a new generation of scientists with interdisciplinary training spanning plant biology, plant genomics, crop breeding environmental sensing, data science, and artificial intelligence will be crucial for the continued growth of enviromics‐assisted breeding. By critically addressing these challenges and proactively pursuing collaborative, interdisciplinary research, abiotic enviromics can fulfill its promise in developing climate‐resilient crops and ensuring global food security in an era of unprecedented environmental change.

## Author Contributions

H.T.N., Y.X., and Y.L. conceptualized the work, G.S. and Y.X. drafted the manuscript, G.S. and Y.X. made the graph. All authors, including H.T.N., J.C.S., J.L.A., and J.X. revised the manuscript with critical comments and suggestions on the draft manuscript, and approved the final manuscript.

## Conflicts of Interest

The authors declare no conflicts of interest.

## Data Availability

No data associated with this manuscript was generated.
